# Impact of conditional deletion of the pro-apoptotic BCL-2 family member BIM in mice

**DOI:** 10.1038/cddis.2014.409

**Published:** 2014-10-09

**Authors:** M J Herold, R Stuchbery, D Mérino, T Willson, A Strasser, D Hildeman, P Bouillet

**Affiliations:** 1 Molecular Genetics of Cancer Division, The Walter and Eliza Hall Institute of Medical Research, Parkville, Victoria, Australia; 2 Department of Medical Biology, The University of Melbourne, Melbourne, Victoria, Australia; 3 Division of Cellular and Molecular Immunology, Department of Pediatrics, Cincinnati Children's Hospital Medical Center, University of Cincinnati, Cincinnati, OH, USA

## Abstract

The pro-apoptotic BH3-only BCL-2 family member BIM is a critical determinant of hematopoietic cell development and homeostasis. It has been argued that the striking hematopoietic abnormalities of BIM-deficient mice (accumulation of lymphocytes and granulocytes) may be the result of the loss of the protein throughout the whole animal rather than a consequence intrinsic to the loss of BIM in hematopoietic cells. To address this issue and allow the deletion of BIM in specific cell types in future studies, we have developed a mouse strain with a conditional *Bim* allele as well as a new Cre transgenic strain, *Vav-CreER*, in which the tamoxifen-inducible CreER recombinase (fusion protein) is predominantly expressed in the hematopoietic system. We show that acute loss of BIM in the adult mouse rapidly results in the hematopoietic phenotypes previously observed in mice lacking BIM in all tissues. This includes changes in thymocyte subpopulations, increased white blood cell counts and resistance of lymphocytes to BIM-dependent apoptotic stimuli, such as cytokine deprivation. We have validated this novel conditional *Bim* knockout mouse model using established and newly developed CreER strains (*Rosa26-CreER* and *Vav-CreER*) and will make these exciting new tools for studies on cell death and cancer available.

The mitochondrial (also called intrinsic, stress or BCL-2 regulated) apoptotic pathway is regulated by members of the BCL-2 family.^1^ This protein family can be divided into pro-survival (A1, MCL-1, BCL-2, BCL-XL and BCL-W) and pro-apoptotic members. The latter can be further sub-divided into the BH3-only (BIM, PUMA, BID, BAD, NOXA, HRK, BMF, BIK) and the multi-BH domain members (BAX, BAK and possibly BOK).^2^ Pro-survival BCL-2 family members protect cells from dying through binding and neutralizing the pro-apoptotic BCL-2 family members. Upon an apoptotic stimulus, the levels of certain BH3-only proteins increase as a result of transcriptional and/or post-transcriptional upregulation. These BH3-only proteins activate the multi-domain members BAX and BAK either directly, or indirectly through neutralizing the pro-survival BCL-2 family members.^3,4^ Activated BAX and BAK oligomerize and form pores into the outer mitochondrial membrane, leading to the release of apoptogenic factors, such as cytochrome *c*, provoking the activation of the so-called caspase cascade with subsequent demolition of the cell.^3^

BIM is a critical initiator of the mitochondrial apoptotic pathway, particularly in hematopoietic cells.^5^ During B- and T-cell development, BIM activity is required to eliminate autoreactive lymphocytes.^6,7^ Constitutive loss of BIM (in all cell types) leads to the accumulation of lymphocytes that infiltrate non-hematopoietic organs, such as lungs, kidneys, liver and salivary glands, produce autoantibodies and on a mixed C57BL/6x129SV background this causes severe autoimmune disease resembling systemic lupus erythematosus.^5^ BIM is also an important factor in peripheral T-cell apoptosis during the shutdown of an immune response.^8,9^ BIM is a tumor suppressor in mantle cell lymphoma, where the gene is lost,^10^ as well as Burkitt's lymphoma and renal carcinoma in which the gene is silenced.^11,12^ Loss of BIM renders cells resistant to several pro-apoptotic stimuli, such as withdrawal of growth factors, treatment with calcium ionophores^5^ or ER stress.^13^

This information about BIM's physiological function was derived from the study of a mouse strain in which the *Bim* gene was constitutively inactivated in all cell types.^5^ The complete absence of a gene product during the entire life of an animal can lead to phenotypes that are not cell-autonomous and may also lead to compensatory events (for example, upregulation of genes with overlapping function). Therefore techniques have been developed so that a gene of interest can be conditionally inactivated in a tissue-specific and/or temporally controllable manner using the Cre/Lox system.^14^ A further refinement of this technique consisted in engineering an inducible Cre recombinase by fusing it with a modified hormone-binding domain of the estrogen receptor (CreER).^15^ In the absence of 4-hydroxytamoxifen (4-OHT), the CreER protein is sequestered in an inactive state in the cytosol. Upon administration of 4-OHT, the ER domain of the CreER fusion protein changes conformation, prompting the translocation of the fusion protein into the nucleus where the recombinase can delete DNA sequences flanked by *loxP* sites.^15^

We developed a conditional *Bim* allele allowing for temporally and spatially controllable deletion of this critical apoptosis initiator. In addition, we developed a novel strain, which expresses the CreER fusion protein under the control of the pan-hematopoietic *Vav* promoter.^16^ We show that the deletion of *Bim* in the adult mouse through activation of the CreER recombinase, using the *Vav-CreER* or the ubiquitously expressed *Rosa-CreER* transgene, caused hematopoietic abnormalities that were similar to those found in the constitutive *Bim* knockout animals. These novel strains (conditional *Bim ko* and *Vav-CreER*) will allow further detailed genetic investigations of cell death and tumourigenesis.

## Results

### BIM expression and phenotype in mice with floxed *Bim* alleles before and after Cre-mediated recombination

**Figure 1:**
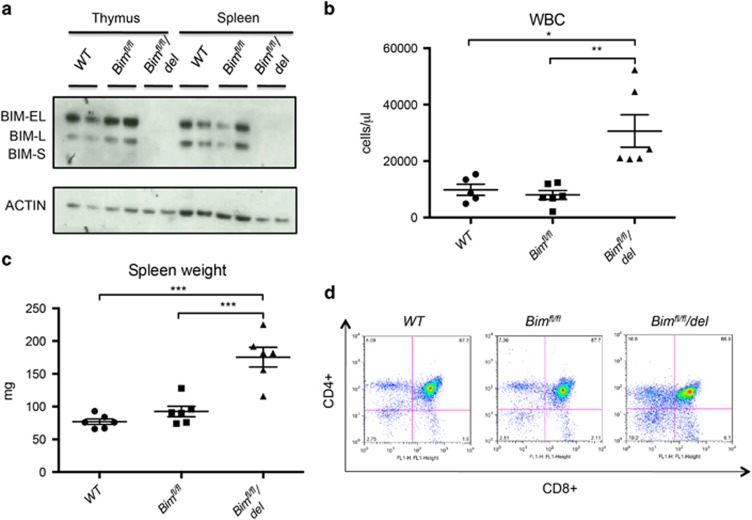
Influence and deletion of a conditional *Bim*^*fl*^ allele in mice. (**a**) Lysates from splenocytes from wt, floxed *Bim* (*Bim*^*fl/fl*^) and BIM-deficient mice (*Bim*^*fl/fl*^*/del*) were immunoblotted for BIM. Probing for *β*-actin served as a loading control. *N*=2 mice per genotype were tested, six independent experiments were performed. (**b**) White blood cell counts and (**c**) spleen weights from wt, *Bim*^*fl/fl*^ and *Bim*^*fl/fl*^*/del* mice were determined. *N*=5–6 mice for each genotype. Data represent mean±S.E.M. *P<0.05, **P<0.01, ***P<0.001 (paired *t*-test). (**d**) Thymocytes from mice of the indicated genotypes were isolated and single-cell suspensions stained with anti-CD4-PE (clone# YTA3.2.1) and anti-CD8-FITC (clone# 53.6.7.2). Dead cells were excluded from analysis by staining with 10 *μ*g/ml propidium iodide (PI; Sigma-Aldrich)

The BH3-only protein BIM is the most critical initiator of apoptosis in hematopoietic cell development and homeostasis.^5^ Mice constitutively deficient for BIM show many abnormalities, including increased white blood cell (WBC) counts, splenomegaly and defects in thymic T-cell selection.^6^ As these defects could potentially be affected by the absence of BIM (i) in non-hematopoietic cells or (ii) be dependent on absence of BIM during embryonic development, we decided to generate mice with a conditional *Bim* allele (*Bim*^*fl*^), to allow specific deletion of *Bim* in a time- and/or tissue-specific manner. Coding exons 2, 3 and 4 of *Bim*^17^ were flanked by *loxP* sites.^18^ As expected, BIM protein expression and hematopoietic cell composition, WBC counts, spleen weights and thymic cell subset distribution, were comparable between *Bim*^*fl/fl*^ and wt mice (Figure 1). Crossing *Bim*^*fl/fl*^ mice with the *CMV-Cre* deleter strain^19^ resulted in the complete loss of BIM protein (*Bim*^*fl/fl*^*/del*; in this strain, Cre is active in the early embryo) (Figure 1a) and the concomitant increase of WBC counts (Figure 1b) and spleen weights (Figure 1c), as well as the altered distribution of thymocyte populations (Figure 1d). These results demonstrate that (i) the *loxP* sequences do not alter the expression of the BIM protein or its function and (ii) that deletion of the floxed *Bim* allele recapitulates the phenotype observed in the constitutive *Bim* knockout mice.

### Induced deletion of *Bim* in adult mice results in phenotypic alterations similar to those observed in constitutive *Bim* knockout mice

**Figure 2:**
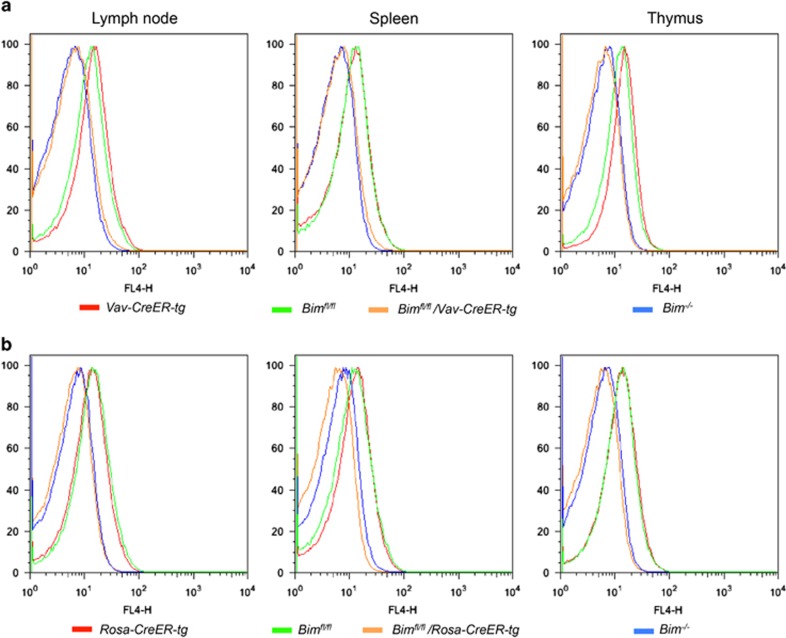
Induced *Bim*^*fl*^ deletion in adult mice. (**a**) *Bim*^*fl/fl*^*/Vav-CreER-tg* and control mice were treated for 5 days and (**b**) *Bim*^*fl/fl*^*/Rosa-CreER-tg* and control mice were treated three times within 5 days (1 day break in between dosing) with a daily dose of 4.8 mg Tamoxifen (4-OHT; Sigma) by oral gavage and then left untreated for 1 month or 3 weeks, respectively. Each FACS plot is representative of *N*>3 repeat experiments

The advantage of a conditional *Bim* allele is the possibility to delete the gene in a temporally and cell type-controllable manner. In order to delete *Bim* at a predetermined time specifically in hematopoietic cells of adult mice, we generated a new transgenic mouse model, in which the tamoxifen-inducible CreERT2 recombinase^20^ is expressed under the control of the pan-hematopoietic *Vav* promoter (*Vav-CreER*).^16^ To induce *Bim* deletion, 12–20-week-old *Bim*^*fl/fl*^*/Vav-CreER-tg* mice were administered 4-OHT by oral gavage. Four weeks after the treatment, lymph nodes, spleens and thymi were collected from 4-OHT-treated *Bim*^*fl/fl*^, *Vav-CreER*, *Bim*^*fl/fl*^/*Vav-CreER* as well as *Bim*^*−/−*^ mice, and BIM protein levels were measured by intracellular FACS analysis (Figure 2a). As expected, the BIM protein levels in cells from 4-OHT-treated *Bim*^*fl/fl*^/*Vav-CreER* mice were comparable to those seen in cells from *Bim*^*−/−*^ mice, whereas the presence of the floxed *Bim* (*Bim*^*fl/fl*^) alleles or *CreER* (*Vav-CreER*) transgene had no impact on BIM protein expression when CreERT2 was not activated. This indicated that both *Bim*^*fl*^ alleles had been successfully recombined in *Bim*^*fl/fl*^*/Vav-CreER* hematopoietic cells of tamoxifen-treated mice, explaining why the BIM protein was no longer present in these cells. To further validate the efficiency of the *Vav-CreER* strain, we crossed this strain to mice harboring a floxed allele of *Mcl-1* (*Mcl1*^*fl*^) and treated *Mcl-1*^*fl/+*^*/Vav-CreER-tg* animals with three doses of 4-OHT. As the deletion of the floxed *Mcl-1* allele leads to the expression of the hCD4 reporter,^21,22^ we analyzed the blood of *Mcl-1*^*fl/+*^*/Vav-CreER-tg* two days post treatment by flow cytometry for hCD4 expression (Supplementary Figure 1). This revealed that ~20% of cells had recombined the floxed *Mcl-1* allele within 1 day, validating the utility of the *Vav-CreER* strain.

We also crossed the *Bim*^*fl/fl*^ mice with the *Rosa-CreER* transgenic strain, in which the ubiquitously expressed CreER protein causes the deletion of *Bim* in the entire organism upon 4-OHT treatment.^23^ Three to four weeks after 4-OHT treatment, BIM protein levels were examined by intracellular FACS analysis. Cells from the lymph nodes, thymus and spleen of *Bim*^*fl/fl*^*/Rosa-CreER-tg* and *Bim*^*−/−*^ mice showed complete absence of the BIM protein, whereas cells from *Bim*^*fl/fl*^ or *Bim*^*+/+*^*/Rosa-CreER-tg* mice expressed similar levels of BIM protein as the corresponding cells from wt mice (Figure 2b). As in the *Rosa-CreER-tg* mice, the *CreER* transgene is ubiquitously expressed, we also analyzed the deletion of the BIM protein in non-haematopoietic tissues (liver, kidney) of 4-OHT-treated *Bim*^*fl/fl*^*/Rosa-CreER-tg* mice. As expected, BIM protein was almost undetectable in these organs, whereas treatment of *Rosa-CreER-tg* mice with 4-OHT had no impact on BIM protein levels (Supplementary Figure 2).

These results demonstrate that recombination of the floxed *Bim* locus can be successfully achieved in the adult mouse using two different 4-OHT-inducible Cre strains (*Vav-CreER* and *Rosa-CreER*).

**Figure 3:**
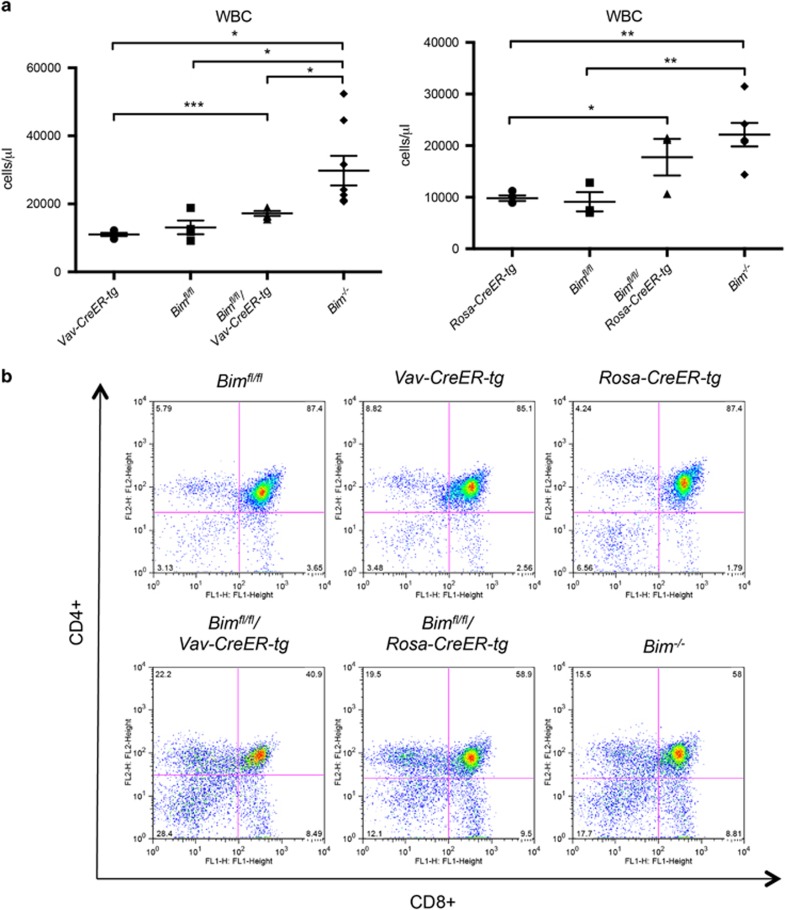
Induced deletion of *Bim*^*fl*^ in mature animals leads to increased WBC numbers and abnormalities in the composition of thymocyte subpopulations. Blood from (**a**) *Bim*^*fl/fl*^*/Vav-CreER-tg* and control mice as well as from (**b**) *Bim*^*fl/fl*^*/Rosa-CreER-tg* and control mice was analyzed with an ADVIA blood analyzer after the animals had been treated with 4-OHT, as described in Figure 2. (**c**) Thymocytes of mice of the indicated genotypes from **a** and **b** were stained with anti-CD4-PE and anti-CD8-FITC and analyzed by flow cytometry. *N*=4–8 mice for each genotype. Data represent mean±S.E.M. *P<0.05, **P<0.01, ***P<0.001 (paired *t*-test)

As loss of BIM in the entire mouse leads to increased WBC numbers,^5^ we analyzed the blood from *Bim*^*fl/fl*^*/Vav-CreER-tg* and *Bim*^*fl/fl*^/*Rosa-CreER-tg* mice 3–4 weeks after 4-OHT treatment (Figure 3). As anticipated, induced loss of BIM caused an increase in WBC numbers in both *Bim*^*fl/fl*^*/Vav-CreER-tg* and *Bim*^*fl/fl*^*/Rosa-CreER-tg* animals, albeit to different levels (Figure 3a). The larger increase observed in the *Bim*^*fl/fl*^*/Rosa-CreER-tg* animals probably reflects a faster deletion of *Bim*^*fl*^ alleles in these animals. 4-OHT-treated *Bim*^*fl/fl*^*/Vav-CreER-tg* and *Bim*^*fl/fl*^*/Rosa-CreER-tg* mice also showed similarly abnormal distribution of thymocyte subpopulations, comparable to what is seen in *Bim*^*−/−*^ mice. This is characterised by abnormally high frequencies of double-negative (CD4^−^CD8^−^) and single-positive (CD4^+^CD8^−^, CD4^−^CD8^+^) thymocytes and reduced proportions of double-positive (CD4^+^CD8^+^) thymocytes compared with control (wt) mice (Figure 3b). This indicates that, although *Bim*^*fl*^ deletion might occur at a slower rate in peripheral lymphoid organs of *Bim*^*fl/fl*^*/Vav-CreER-tg* mice compared with the *Bim*^*fl/fl*^*/Rosa-CreER-tg* animals, *Bim*^*fl*^ recombination in thymocytes (or their precursors) appears to occur at similar rates in both strains.

### Induced deletion of *Bim* in the adult mouse protects thymocytes from BIM-dependent apoptotic stimuli

**Figure 4:**
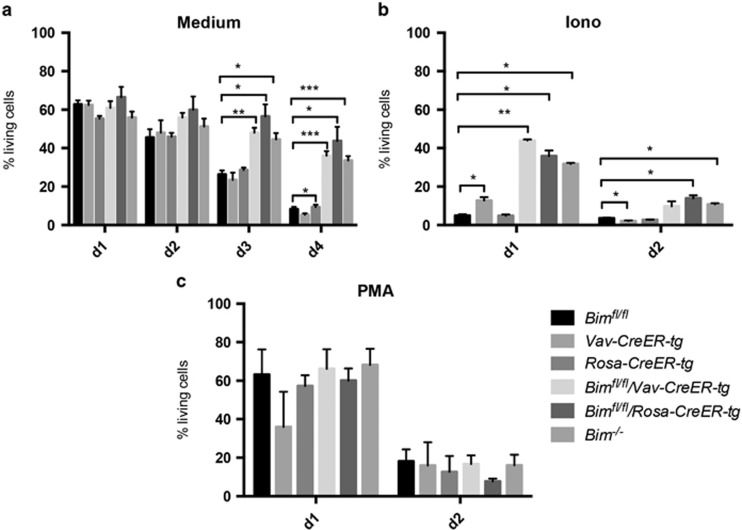
Induced deletion of *Bim*^*fl*^ in adult animals renders thymocytes resistant to BIM-dependent apoptotic stimuli. Thymocytes from 4-OHT-treated *Bim*^*fl/fl*^*/Vav-CreER-tg*, *Bim*^*fl/fl*^*/Rosa-CreER-tg* and control mice were cultured (**a**) in medium only, or were (**b**) treated with 1 *μ*g/ml Ionomycin (Sigma) or (**c**) with 2 ng/ml PMA (Sigma). At the indicated time points, the percentages of live cells were determined by staining with AnnexinV/PI (AnnexinV-PI=living cells). *N*=3–7 mice for each genotype. Data represent mean±S.E.M. *P<0.05, **P<0.01, ***P<0.001 (paired *t*-test)

BIM-deficient thymocytes are resistant to a variety of pro-apoptotic stimuli.^5^ We therefore compared the response of thymocytes from *Bim*^*−/−*^, *Bim*^*fl/fl*^*/Vav-CreER-tg*, *Bim*^*fl/fl*^*/Rosa-CreER-tg*, *Bim*^*fl/fl*^, *Vav-CreER* and *Rosa-CreER* mice, which had been treated with 4-OHT four weeks prior to organ isolation, to diverse cytotoxic stimuli. Thymocytes of the different genotypes were cultured in medium (medium; mimicking cytokine withdrawal) or treated with BIM-dependent (Ionomycin=Iono) and BIM-independent (phorbol ester=PMA) apoptotic stimuli (Figure 4). Although thymocytes from mice of all genotypes were killed at a similar rate when treated with PMA (kills in a PUMA-dependent manner^24^), untreated (medium) and Ionomycin-treated thymocytes from 4-OHT-treated *Bim*^*−/−*^, *Bim*^*fl/fl*^*/Vav-CreER-tg* and *Bim*^*fl/fl*^*/Rosa-CreER-tg* mice all showed a marked survival advantage when compared with thymocytes from 4-OHT-treated *Bim*^*fl/fl*^, *Rosa-CreER-tg* and *Vav-CreER-tg* mice. This demonstrates that constitutive or induced deletion of *Bim* in thymocytes leads to a similar resistance to these apoptotic stimuli, and that the observed change in thymocyte distribution is a consequence of the loss of BIM and not due to other abnormalities caused by Cre-mediated recombination.

## Discussion

We report here the development of a new *floxed Bim* allele, which allows the deletion of this pro-apoptotic BH3-only protein in a cell type-restricted and temporally controllable manner. Using the well-established *Rosa-CreER* and newly developed *Vav-CreER* transgenic strains, deletion of *Bim* in the adult mice results in the same phenotype as that observed in the constitutive *Bim* knockout mice. It thus appears that the hematopoietic phenotype associated with the constitutive loss of BIM in all cell types reported previously^5^ is intrinsic to the hematopoietic system rather than a consequence of an unrecognized developmental defect due to the absence of BIM.

Our studies clearly show that the floxed *Bim* allele is functional and can be efficiently deleted by CreER recombinases. Importantly, these *Bim*^*fl/fl*^ mice have recently been used in two other studies,^18,25^ in which *Bim* was specifically deleted in the regulatory T-cell lineage (Treg) only. This new mouse strain will thus be a valuable tool to further dissect the role of BIM in various cell types.

Importantly, our new *Vav-CreER* transgenic strain showed the same deletion efficiency of the floxed *Bim* alleles 4 weeks after 4-OHT treatment as the very well-characterised and widely used *Rosa-CreER* strain.^23^ However, when we tested the loss of BIM protein 5 days after 4-OHT treatment, we observed residual BIM protein in the lymphoid cells from the *Bim*^*fl/fl*^/*Vav-CreER* mice, whereas it was almost completely absent in the cells from the *Bim*^*fl/fl*^/*Rosa-CreER* animals at this time point (data not shown). This indicates that deletion of *Bim*^*fl*^ alleles occurs more rapidly and probably also more efficiently in the *Bim*^*fl/fl*^/*Rosa-CreER* strain than in the *Bim*^*fl/fl*^/*Vav-CreER* strain, possibly because of the lower expression of CreER in the *Vav-CreER* strain.

However, the higher efficiency of Cre-mediated recombination of floxed target genes in the *Rosa-CreER* strain is accompanied by a higher toxicity observed in the animals upon treatment with 4-OHT. Accordingly, treatment of *Rosa-CreER* or *Vav-CreER* transgenic mice with 4-OHT for 5 consecutive days led to substantial destruction of lymphoid organs in the former, whereas these organs remained unaffected in the latter (Supplementary Figure 3 and data not shown). Both CreER strains are highly efficient at recombining the floxed *Bim* allele and, depending on the experimental requirements, are valuable tools for induced deletion of floxed alleles in mature or developing animals.

A significant leakage in CreER recombinase activity was observed in the *Bim*^*fl/fl*^*/Rosa26-CreER* strain, as tail DNA obtained at weaning showed evidence of Cre-mediated deletion of the floxed *Bim* allele in ~20% of these animals (data not shown). This was never observed in *Bim*^*fl/fl*^*/Vav-CreER-tg* mice. Note that only mice with no evidence of *Bim*^*fl*^ recombination prior to tamoxifen administration were used in this study. Pertinently, we observed no abnormalities in splenic weights and thymocyte sub-population distribution in *Bim*^*fl/fl*^*/Vav-CreER-tg* and *Bim*^*fl/fl*^*/Rosa-CreER-tg* animals prior to 4-OHT treatment (data not shown). We therefore conclude that the occasional leakiness of CreER recombinase activity in the *Rosa-CreER-tg* mice must be limited to embryonic development and that despite this issue (which can be identified by tail DNA analysis), both this inducible CreER mouse strain and the *Vav-CreER-tg* strain are suitable tools for the inducible deletion of floxed genes.

## Materials and Methods

### Mice

Experiments with mice were conducted according to the guidelines of The Walter and Eliza Hall Institute Animal Ethics Committee. The generation of the conditional *Bim*^*fl*^, *Mcl-1^fl^* and the *RosaCreER* mice, all on a C57BL/6 background, has been described previously.^18,21,26^ *Vav-CreER* transgenic mice were generated by replacing the hCD4 sequence of the *Vav* hematopoietic vector^27^ with the *CreERT2*^20^ sequence using *Sfi*1/*Not*1 restriction sites. The *Vav-CreER* construct was linearized with *Hin*dIII before pro-nuclear injection of the DNA into zygotes derived from C57BL/6 mice. Positive offspring were identified by PCR for the genomic integration of the *Vav-CreER* transgene.

To activate the latent CreER recombinase, mice were given 200 mg/kg tamoxifen (Sigma-Aldrich, Rowville, VIC, Australia) in peanut oil/10% ethanol each day for 5 days by oral gavage.^28^

### Western blotting and WBC analysis

Cell extracts for western blot analysis were prepared in ONYX lysis buffer (20 mM Tris-HCl pH 7.4, 135 mM NaCl, 1.5 mM MgCl_2_, 1 mM EGTA, 1% Triton X-100, 10% glycerol). Antibodies used include: rat anti-BIM (clones 3C5 and CF7, ENZO Life Sciences,^29^ Waterloo, NSW, Australia), mouse anti-*β*-actin (Sigma AC-40, Rowville, VIC, Australia). Horseradish peroxidase-conjugated goat anti-mouse IgG or goat anti-rat IgG antibodies (both from Southern Biotech, Birmingham, AL, USA) served as secondary reagents and the enhanced chemoluminescence (ECL; GE Healthcare, Rydalmere, NSW, Australia) system was used for detection. WBC analysis was performed with an ADVIA blood analyzer (Siemens Healthcare Diagnostics, Tarrytown, NY, USA).

### Intracellular immunofluorescent staining of BIM for flow cytometric analysis

Cells (1 × 10^6^) from lymph nodes, spleen and thymus were isolated from mice of the indicated genotypes, fixed and permeabilized by suspension in 100 *μ*l of BD Cytofix/Cytoperm (BD Biosciences, San Jose, CA, USA) solution for 20 min on ice. Cells were then washed twice in Perm/Wash buffer (BD Biosciences) and stained with Alexa-647-conjugated BIM antibody (rat, clone # 3C5,^29^ ENZO Life Sciences). Analysis was performed in a FACSalibur (BD Biosciences).

### Statistical analysis

Statistical comparisons were made using a two-tailed Student's *t*-test with Prism v.5.0 (GraphPad Software, Inc., La Jolla, CA, USA). *P*-values <0.05 were considered to indicate a statistically significant difference. *n* indicates the number of mice analyzed for each genotype.

## Abbreviations

4-OHT: 4-hydroxytamoxifen
WBC: white blood cells
PMA: phorbol ester
Iono: ionomycin
Treg: regulatory T cells
Tg: transgenic
