# Supplementary material for: Impact of conditional deletion of the pro-apoptotic BCL-2 family member BIM in mice
Source: Cell Death Dis. 2014 Oct 9;5(10):e1446–. doi: 10.1038/cddis.2014.409 (PMC4237241; doi:10.1038/cddis.2014.409)
Supplement: Supplementary Figure Legends [file cddis2014409x1.doc]

***Supplementary Figures:***

**Supplementary Figure1.** Efficiency of the recombination of floxed genes in the *Vav-CreER* mice.  *Mcl-1^fl/+^/Vav-CreER-tg*, *Vav-CreER-tg* and *Mcl-1^fl/+^* mice were treated with three doses of 4-OHT (200 mg/kg) by oral gavage. Three days later blood cells were stained with antibodies to human CD4 (clone RPA-T4) and analysed by flow cytometry.

**Supplementary Figure 2.** *Bim* deletion in non-haematopoietic cells of the *Bim^fl/fl^*/*Rosa-CreER* mice*. Bim^fl/fl^*/*Rosa-CreER-tg* or *Rosa-CreER-tg* mice were treated with 3 doses of 200 mg/kg 4-OHT over the course of 5 days. 4 weeks later protein lysates from liver and kidney were generated. Western blot analysis for BIM protein was performed. Probing for HSP70 served as a loading control.

**Supplementary Figure 3.** Toxicity analysis in *Rosa-CreERtg* mice*.* Mice of the indicated genotypes were treated for 5 consecutive days with 4-OHT (200 mg/kg). Five days later, the blood of these mice was analysed by the Advia Blood analyser and splenic and thymic cell numbers were also determined. The data represent means +/- SD; *p<0.05; ** p<0.01. n = 3 – 5 mice.
