# Supplementary figures and images for: Impact of conditional deletion of the pro-apoptotic BCL-2 family member BIM in mice
Source: Cell Death Dis. 2014 Oct 9;5(10):e1446–. doi: 10.1038/cddis.2014.409 (PMC4237241; doi:10.1038/cddis.2014.409)

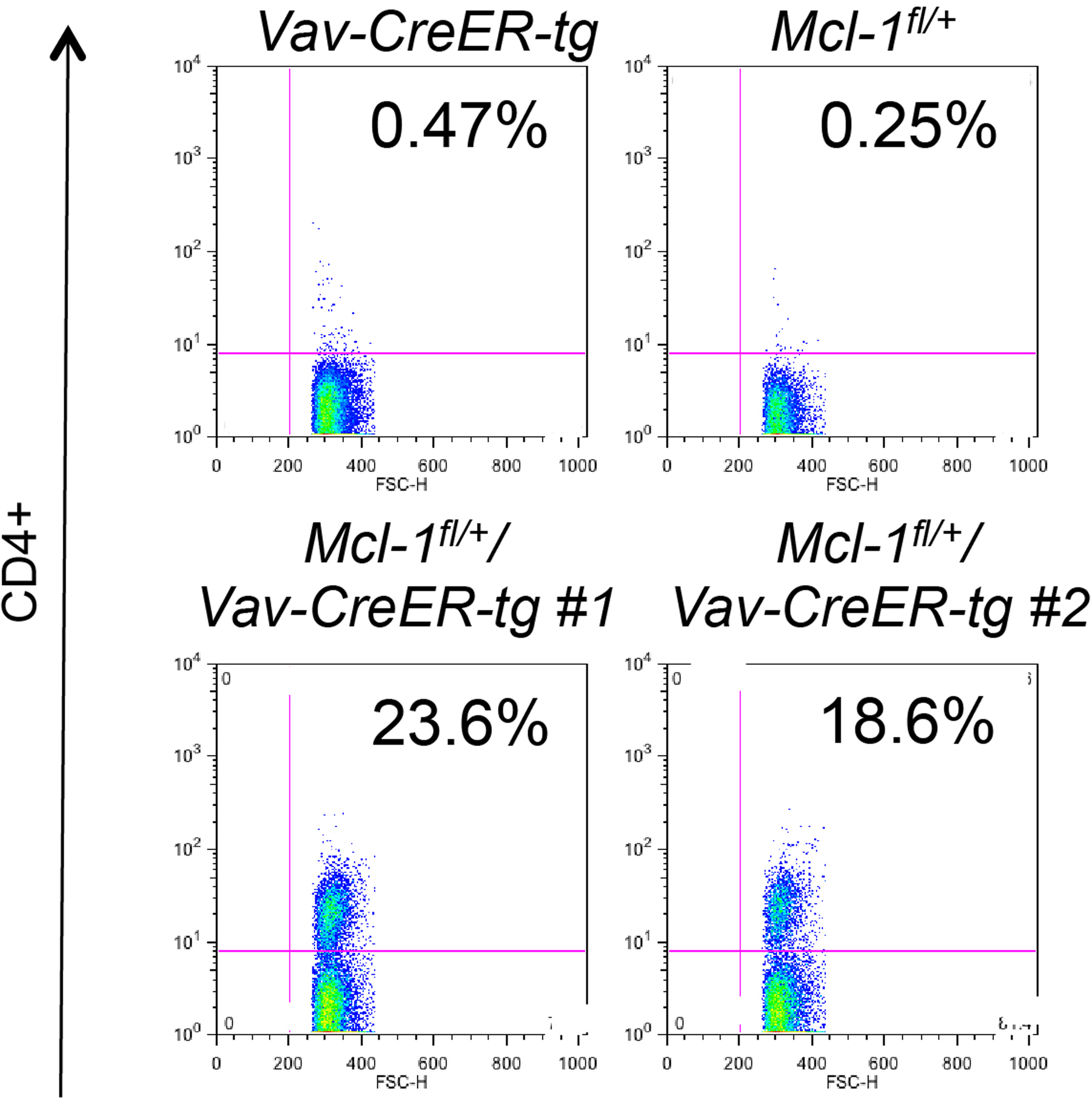

Supplement: Supplementary Figure 1 [file cddis2014409x2.tif]

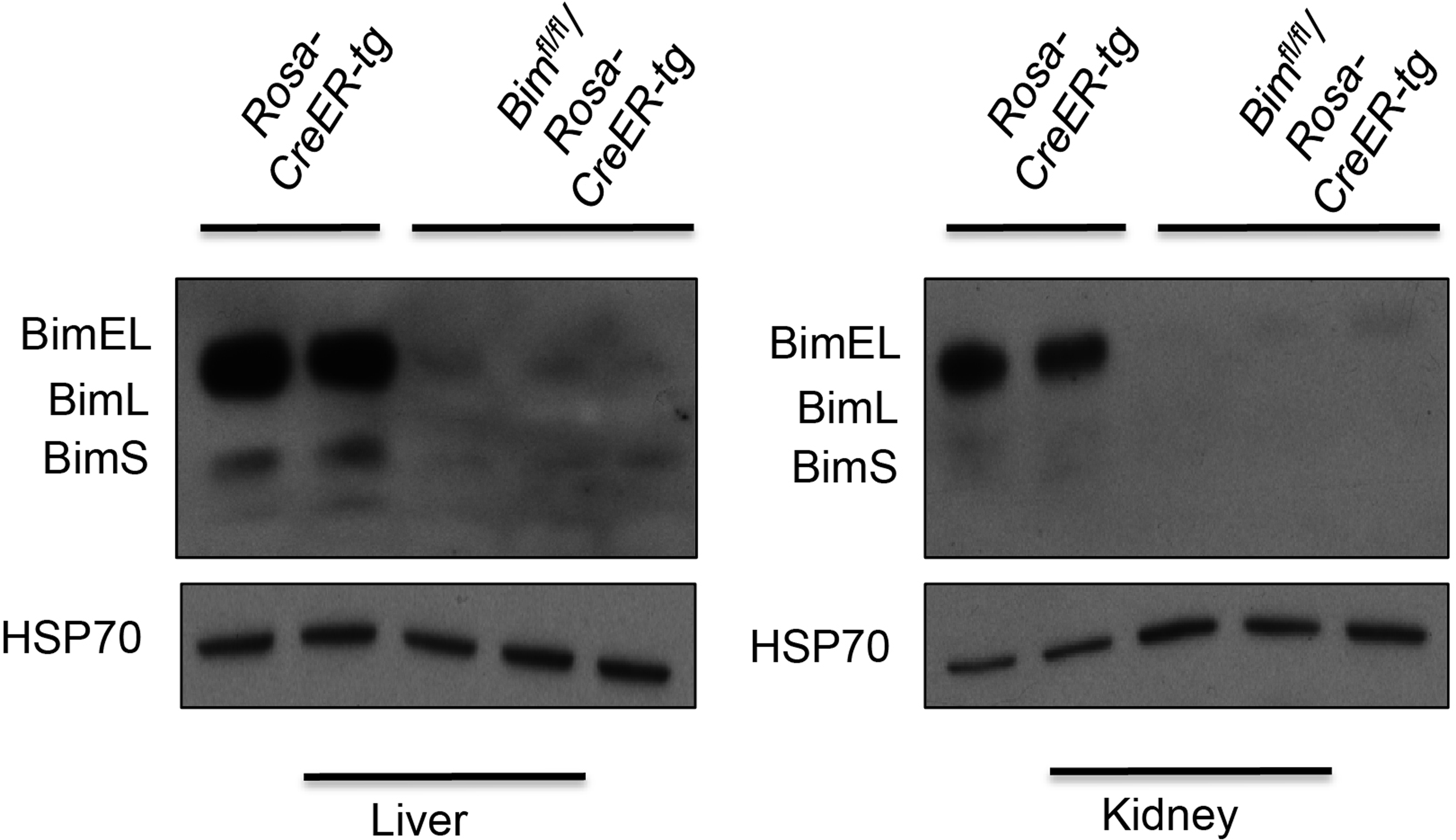

Supplement: Supplementary Figure 2 [file cddis2014409x3.tif]

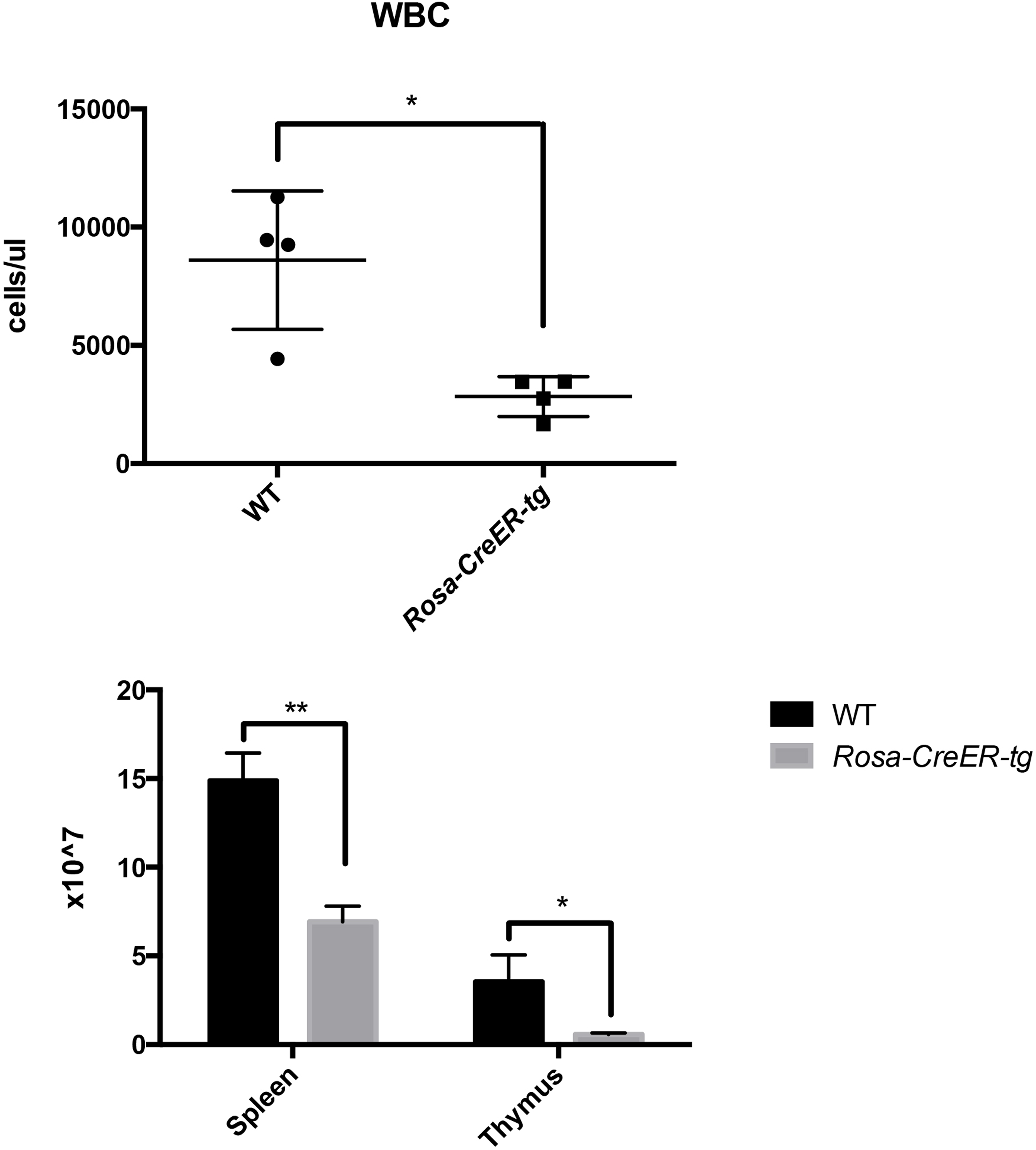

Supplement: Supplementary Figure 3 [file cddis2014409x4.tif]
